# Assessment of inflammatory markers and their association with disease mortality in severe COVID-19 patients of tertiary care hospital in South India

**DOI:** 10.1186/s43168-022-00159-1

**Published:** 2022-11-04

**Authors:** Nayana Devang, Souparnika Sreelatha, Mamatha B. V.

**Affiliations:** 1 Department of Biochemistry, Kanachur Institute of Medical Sciences and Research Centre, Mangalore University Road, Deralakatte, Mangaluru, Karnataka 575018 India; 2 Department of Biochemistry, KS Hegde Medical Academy, NITTE University, Deralakatte, Mangaluru, Karnataka 575018 India

**Keywords:** COVID-19, Inflammation, C-reactive protein, Lactate dehydrogenase, Ferritin, Oxygen saturation, Mortality

## Abstract

**Background:**

COVID-19 infection involves a complex interplay of the immunological and inflammatory responses. Low blood-oxygen levels have been a hallmark in COVID-19 patients. The lung tissue damage infiltered by the viral-mediated inflammation decreases oxygen saturation to cause silent hypoxia and cell death. This study aimed to evaluate the association of inflammatory biomarkers with oxygen saturation (SpO_2_) and mortality in severe COVID-19 patients.

**Methods:**

A total of 190 severe COVID-19 patients were included in this study after confirmed by the RT-PCR assay. The laboratory tests were performed for biochemical assessment. Serum levels of C-reactive protein (CRP), lactate dehydrogenase (LDH), and ferritin were determined and compared between survivors and nonsurvivors using independent sample *t*-test. The correlation of these inflammatory markers was studied using Spearman’s correlation, and their association with mortality was studied using logistic regression.

**Results:**

All the COVID-19 patients were severe with SpO_2_< 90% and respiratory rate > 30/min. While the serum levels of CRP, LDH, ferritin, aspartate transaminase (AST), urea, and random blood sugar (RBS) were elevated, hemoglobin (Hb) and SpO_2_ levels were reduced in COVID-19 patients. LDH and ferritin levels were significantly higher in nonsurvivors compared to survivors with *p* values of 0.001 and 0.022 respectively. Spearman’s correlation showed a significant correlation of the inflammatory markers with SpO_2_, serum electrolytes (potassium, chloride), liver enzymes (AST and alanine transaminase (ALT)), and markers of kidney damage (urea, creatinine).

**Conclusion:**

Inflammatory markers could effectively discriminate the risk of mortality in severe COVID-19 patients. As CRP, LDH, and ferritin levels determine the tissue oxygen availability, they seem to be valuable biomarkers in the prognosis of COVID-19.

## Background

A novel coronavirus, 2019-nCoV, that has been identified to have originated in Wuhan, China, has evolved into a pandemic, and as of today, more than 500 million people have been infected and more than six million have died globally.

COVID-19 inflammation has been linked to disease severity [1]. Accumulative evidence has demonstrated that cytokine storm by immune cells during the cell lysis stage of COVID-19 viral replication raises C-reactive protein (CRP) and lactate dehydrogenase (LDH) levels [2–5]. It has been demonstrated that the mortality is due to the severe multisystemic end-organ failure as a result of cytokine storm. Hence, measuring the inflammatory markers prove important for prognostication and management of these patients. CRP produced in a liver has been reported to be significantly associated with the higher risks of the COVID-19 infection. The proportion of patients with elevated CRP levels was significantly higher in severe COVID-19 patients than in nonsevere patients and CRP levels in the early stages correlates with the disease severity [6, 7], CRP can act as an early marker of COVID-19 infection and inflammation which could help health workers to enable earlier clinical intervention in high-risk population [8].

Pooled analysis by Henry et al. reported that LDH levels predict COVID-19 severity. Elevated LDH levels associated with 6-fold increase in odds of developing severe covid disease and 16-fold increase in odds of mortality in COVID-19 patients [9]. While fewer studies have reported the predictive role of CRP in COVID-19 mortality, there is a dearth in the study of the association of LDH and ferritin with mortality among severe COVID-19 patients. Hence, we conducted a cohort (survivors-non-survivors) study of ICU-admitted COVID-19 patients to study the association between inflammation and mortality.

## Methods

### Study design

A cross-sectional study was carried out on severe COVID-19 patients admitted to the tertiary care hospital attached to Kanachur Institute of Medical Sciences, Mangaluru, in January 2022. This study has been approved by the Institutional ethics committee.

### Subjects

Sample size was calculated at 95% confidence interval, with a power of 80% based on the study of Szarpac et al. [10] using the formula:

$$n=2\left({\left({Z}_{\alpha }+{Z}_{\beta}\right)}^2\ {\upsigma}^2\right)/{d}^2$$

A total of 190 COVID-19 patients were included in the study. SARS-CoV-2 infection was confirmed by the RT-PCR assay conducted in accordance with the standard protocol. All the COVID-19 patients were severe with SpO_2_ <90% on room air at sea level and respiratory rate >30/min [11]. Patients with cancer; with pre-existing musculoskeletal disease; with chronic diseases of liver, kidney, and heart; with bacterial and non-covid viral infections; and on immunosuppressive drugs for another disease and pregnant and lactating women were excluded.

### Samples

In plain vacutainer, 5 ml of venous blood was collected and allowed to clot for 30 min at room temperature and then centrifuged at 2400×g for 10 min to separate serum. The analysis of inflammatory markers (CRP, LDH, ferritin), SpO_2_, liver markers (AST, ALT, ALP), markers of kidney function (electrolytes, urea, creatinine), and random blood sugar (RBS) was performed.

### Methods

Tests for biochemical parameters were performed on the VITROS 5600/XT 7600 Integrated Systems according to the manufacturer’s instructions. CRP was determined based on the principle of the latex agglutination. LDH was determined based on the principle of the enzymatic coupling reaction. LDH catalyzes the conversion of pyruvate and NADH to lactate and NAD^+^. Oxidation of NADH was monitored by reflectance spectrophotometry, which is used to measure the LDH activity. The ferritin was measured using the principle of immuno-turbidimetry. Agglutination formed due to the reaction between latex-bound ferritin antibodies and the antigen in the sample to form an antigen/antibody complex was measured turbidometrically.

### Statistical analysis

SPSS 20.0 (SPSS Inc., Chicago, IL, USA) was utilized for statistical analysis. Values are expressed as mean ± standard deviation. The one-sample *t*-test was used to determine the significant difference in the mean values of inflammatory markers from reference value. Independent sample *t*-test was used to compare the values between survivors and nonsurvivors. To know the correlation of these inflammatory markers, Spearman’s correlation was done. To know the association of inflammation with mortality, logistic regression was performed. Receiver operating characteristic (ROC) curves were used to obtain the optimal probability cut-off to predict the mortality. A *p* value of <0.05 was considered significant.

## Results

A total of 190 severe COVID-19 patients were included in this study; 105 patients were males and 85 patients were females. All the COVID-19 patients were severe with SpO_2_ <90% on room air at sea level. Seventy-five percent (*N* = 142) of patients were survivors and 25% (*N* = 48) of patients were nonsurvivors. The mean age of the participants was 53 years. There was no significant difference in age between males and females.

The laboratory findings of the studied patients at the time of diagnosis are presented in Table 1. While the levels of CRP, LDH, ferritin, AST, ALT, urea, RBS, and SBP were elevated, the level of hemoglobin and SpO_2_ were reduced in COVID-19 patients. The significant differences in the mean values of biochemical parameters between males and females are mentioned in Table 2. Serum ferritin, urea, and creatinine values were significantly higher in male COVID-19 patients than female patients (*p* < 0.05).

**Table 1:** Biochemical parameters of the study participants

Parameters	Mean ± SD
No. of participants (*N*)	190
Age (years)	53 ± 14.68
Sodium (mEq/L)	136.16 ± 5.041
Potassium (mEq/L)	4.26 ± 0.85
Chloride (mEq/L)	100 ±17.56
LDH (U/L)	511.12 ±328.75
CRP (mg/L)	136.94 ±118.77
Ferritin (ng/ml)	721.42 ± 892.14
AST (U/L)	90.07 ± 282.79
ALT (U/L)	63.75 ± 117.68
Urea (mg/dl)	55.07 ± 46.71
Creatinine (mg/dl)	1.37 ± 2
ALP (IU/L)	121.95 ± 80.4
RBS (mg/dl)	219.03 ± 127.88
SpO_2_ (%)	63.21 ± 19.68
Hb (gm/dl)	12.3 ± 1.92
SBP (mmHg)	136.06 ± 19.51
DBP (mmHg)	82.82 ± 10.36

*LDH* lactate dehydrogenase, *CRP* C-reactive protein, *AST* aspartate transaminase, *ALT* alanine transaminase, *ALP* alkaline phosphatase, *RBS* random blood sugar, *SpO*_*2*_ oxygen saturation, *Hb* hemoglobin, *SBP* systolic blood pressure, *DBP* diastolic blood pressure

**Table 2:** Comparison of biochemical parameters between male and female covid participants

Parameters	Males Mean ± SD	Females Mean ± SD	*p* value
*N*	105	85	NA
Age (years)	56 ± 14.254	53 ± 14.28	0.317
Sodium (mEq/L)	136.82 ± 6.24	136.96 ± 5.15	0.446
Potassium (mEq/L)	4.23 ± 0.935	4.05 ± 0.696	1.139
Chloride (mEq/L)	102.94 ± 17.78	99.86 ± 6.16	0.105
LDH (U/L)	570.03 ± 400.95	455.53 ± 211.295	0.062
CRP (mg/L)	154.74 ± 118.85	118.32 ± 116.83	0.301
Ferritin (ng/ml)	1280.55 ± 1481.86	557.97 ± 724.12	<0.001^**^
AST (U/L)	70.43 ± 44.93	95.63 ± 347.19	0.077
ALT (U/L)	54.16 ± 50.92	63.76 ± 138.67	0.074
Urea (mg/dl)	64.79 ± 48.18	45.55 ±37.17	0.005^*^
Creatinine (mg/dl)	1.84 ± 2.59	1.16 ± 1.11	0.023^*^
ALP (IU/L)	130.32 ± 82.66	117.19 ± 65.15	0.213
RBS (mg/dl)	149.57 ± 100.18	171.6 ± 144.25	0.345
SpO_2_ (%)	62.3 ± 21.24	64.42 ± 17.57	0.076
Hb (gm/dl)	12.87 ± 2.13	11.81 ± 1.56	0.093
SBP (mmHg)	136.94 ± 20.15	135.02 ± 18.87	0.823
DBP (mmHg)	82.82 ± 10.96	82.82 ± 9.7	0.733

*LDH* lactate dehydrogenase, *CRP* C-reactive protein, *AST* aspartate transaminase, *ALT* alanine transaminase, *ALP* alkaline phosphatase, *RBS* random blood sugar, *SpO*_*2*_ oxygen saturation, *Hb* hemoglobin, *SBP* systolic blood pressure, *DBP* diastolic blood pressure

**p value <0.001, *p value <0.05

The results of one sample *t* test showed the levels of LDH, CRP, and ferritin to be significantly higher than the population mean with *p* value less than 0.001 (Table 3). LDH and ferritin levels were significantly higher in nonsurvivors compared to survivors with *p* values of 0.001 and 0.022 respectively (Table 4). There was no significant difference in CRP between the two groups. Other parameters that were significantly higher in nonsurvivors compared to survivors were potassium, urea, and creatinine (Table 4).

**Table 3:** Results of one sample *t* test to compare the serum levels of inflammatory markers in covid patients against the population mean

Inflammatory markers	Mean ± SD	One sample *t* test *t* value	*p* value
LDH (U/L)	517.6 ±331.587	7.412	<0.001^**^
CRP (mg/L)	136.95 ±118.821	12.804	
Ferritin (ng/ml) males	1280.55 ± 1481.86	6.374	
Ferritin (ng/ml) females	557.97 ± 724.12	4.65	

*LDH* lactate dehydrogenase, *CRP* C-reactive protein

**p value <0.001

**Table 4:** Comparison of biochemical parameters between survivors and nonsurvivors

Parameters	Survivors Mean ± SD	Nonsurvivors Mean ± SD	*p* value
*N*	142	48	NA
Age (years)	51 ± 14.68	58 ± 13.6	0.282
Sodium (mEq/L)	135.99 ± 4.99	136.46 ± 5.15	0.634
Potassium (mEq/L)	3.96 ± 0.62	4.65 ±1.06	<0.001^**^
Chloride (mEq/L)	100.4 ±21.76	99.39 ± 6.4	0.094
LDH (U/L)	408.52 ± 182.56	675.28 ± 432.06	0.001^*^
CRP (mg/L)	112.42 ± 104.92	181.22 ± 130.33	0.095
Ferritin (ng/ml)	544.07 ± 762.89	999.43 ± 1013.22	0.022^*^
AST (U/L)	54.92 ± 44.88	178.55 ± 521.49	0.213
ALT (U/L)	49.11 ± 50.59	100.62 ± 203.48	0.188
Urea (mg/dl)	36.43 ± 27.501	80.59 ± 55.16	<0.001^**^
Creatinine (mg/dl)	0.91 ± 0.64	2.36 ± 3.05	0.002^*^
ALP (IU/L)	121.79 ± 82.67	122.28 ± 76.74	0.822
RBS (mg/dl)	200.78 ± 117.84	253.91 ± 139.99	0.26
SpO_2_ (%)	68.05 ± 19.898	56.26 ± 17.35	0.103
Hb (gm/dl)	12.34 ± 1.78	12.36 ± 2.21	0.207
SBP (mmHg)	131.96 ± 16.57	145.61 ± 22.57	0.335
DBP (mmHg)	82.12 ± 10.15	84.44 ± 10.81	0.847

*LDH* lactate dehydrogenase, *CRP* C-reactive protein, *AST* aspartate transaminases, *ALT* alanine transaminase, *ALP* alkaline phosphatase, *RBS* random blood sugar, *SpO*_*2*_ oxygen saturation, *Hb* hemoglobin, *SBP* systolic blood pressure, *DBP* diastolic blood pressure

**p value <0.001, *p value <0.05

Spearman’s correlation showed a significant correlation of the inflammatory markers with each other and with serum electrolytes such as potassium and chloride, liver enzymes such as AST and ALT, markers of kidney damage such as serum urea and creatinine, and SpO_2_ (Table 5). Ferritin also showed a positive correlation with ALP. While there was no correlation of CRP with SpO_2_, LDH and ferritin showed negative correlation with SpO_2_ (Table 5).

**Table 5:** Correlation of inflammatory markers with different biochemical parameters

A. Correlation of inflammatory markers with markers of liver function						
	LDH	CRP	Ferritin	AST	ALT	ALP
LDH			*r* = 0.509 *p* ≤ 0.001	*r* = 0.545 *p* ≤ 0.001	*r* = 0.299 *p* = 0.006	
CRP	*r* = 0.285 *p* = 0.003		*r* = 0.23 *p* = 0.025	*r* = 0.245 *p* = 0.006	*r* = − 0.283 *p* = 0.01	
Ferritin	*r* = 0.509 *p* < 0.001	*r* = 0.23 *p* = 0.025		*r* = 0.352 *p* < 0.001	*r* = 0.203 *P*= 0.026	*r* = 0.260 *p* = 0.005
B. Correlation of inflammatory markers with markers of kidney function						
	K	Cl	Na	Urea	Creatinine	
LDH	*r* = 0.212 *p* = 0.031			*r* = 0.210 *p* = 0.045	*r* = 0.316 *p* = 0.001	
CRP	*r* = 0.245 *p* = 0.006	*r* = − 0.283 *p* = 0.01		*r* = 0.373 *p* < 0.001	*r* = 0.354 *p* < 0.001	
Ferritin	NA			*r* = 0.371 *p* < 0.001	*r* = 0.277 *p* = 0.001	
C. Correlation of inflammatory markers with blood sugar and oxygen saturation						
	RBS	SpO_2_				
LDH		*r* = − 0.302 *p* = 0.004				
CRP	*r* = 0.2 *p* = 0.026	NA				
Ferritin		*r* = − 0.374 *p* = 0.001				

*LDH* lactate dehydrogenase, *CRP* C-reactive protein, *AST* aspartate transaminase, *ALT* alanine transaminase, *ALP* alkaline phosphatase, *RBS* random blood sugar, *SpO*_*2*_ oxygen saturation

Further evaluation of the association between inflammatory markers (CRP, LDH, and ferritin) and COVID-19 mortality was performed by Logistic Regression Analysis. CRP had OR of 1.005 (95% CI= 1.002–1.008, *p* < 0.002). LDH had OR of 1.004 (95% CI= 1.002–1.006, *p* < 0.001). Ferritin had OR of 1.001 (95% CI = 1–1.001, *p* = 0.028) (Table 6).

**Table 6:** CRP, LDH, and ferritin as a predictor of COVID-19 mortality

Inflammatory markers	Odds ratio (95% CI)	*p* value
CRP	1.005 (1.002–1.008)	0.002
LDH	1.004 (1.002–1.006)	<0.001
Ferritin	1.001 (1–1.001)	0.028

*LDH* lactate dehydrogenase, *CRP* C-reactive protein

**Fig. 1:**
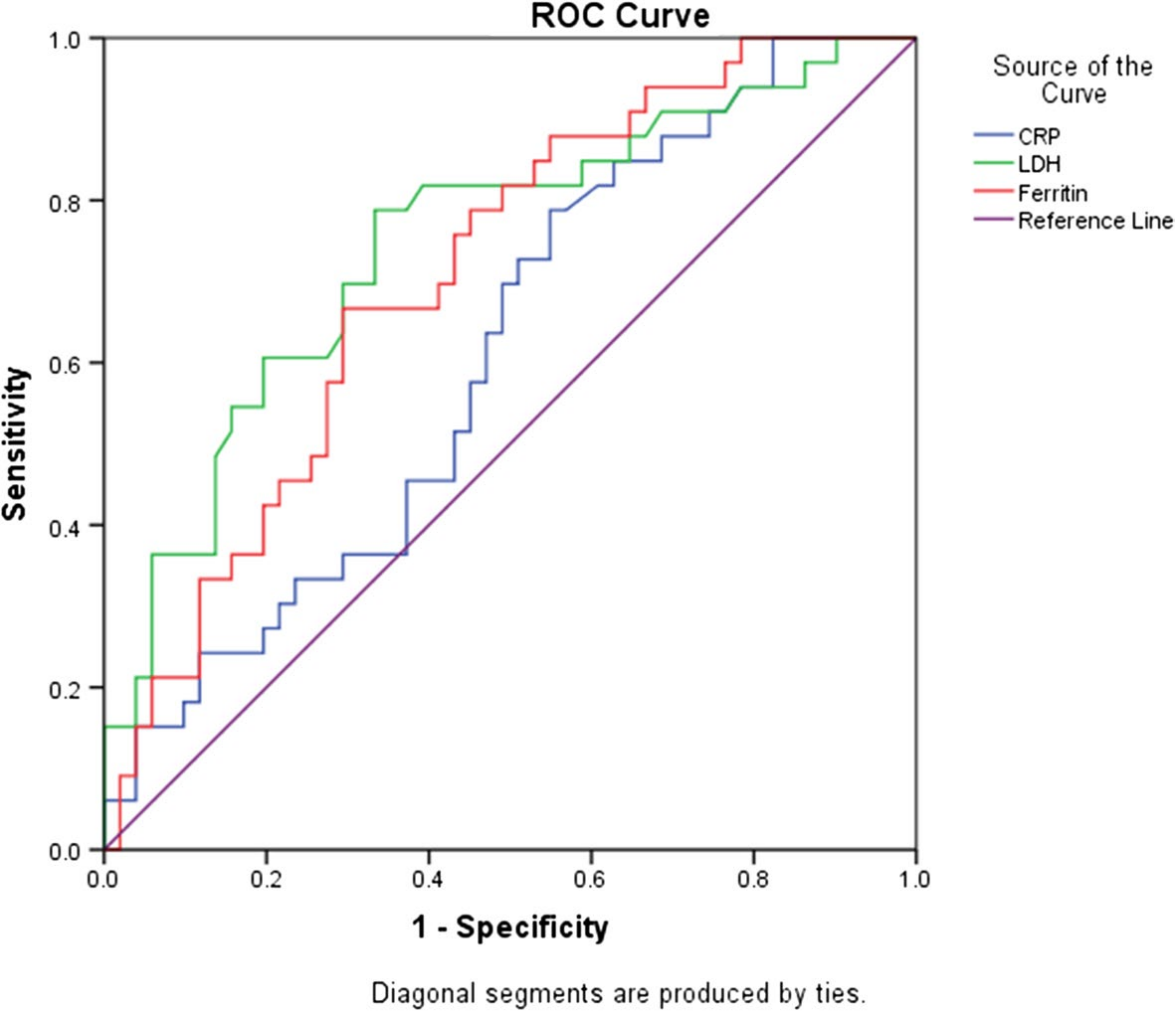
ROC curve of inflammatory markers as predictors of COVID-19 mortality

The role of inflammatory markers in predicting the COVID-19 mortality was evaluated using the ROC curve. Survivors were considered negative and severe patients as positive. The area under the ROC curve (ROC-AUC) was assessed (Fig. 1).

COVID-19 mortality was assessed by ROC curve analysis. The ROC-AUC for CRP, LDH, and ferritin were 0.610, 0.748, and 0.709 respectively. LDH and ferritin are the sensitive predictors of COVID-19 mortality (Fig. 1, Table 7).

**Table 7:** The performance criteria of the inflammatory markers in predicting COVID-19 mortality

Inflammatory markers	Cutoff	Sensitivity	Specificity	AUC (95% CI)	*p* value
CRP	>147.5	0.545	0.549	0.610 (0.489–0.731)	0.09
LDH	>433	0.7	0.667	0.748 (0.638–0.857)	<0.001
Ferritin	>489	0.667	0.647	0.709 (0.599–0.819)	0.001

## Discussion

This study provided the baseline laboratory characteristics of severe COVID-19 patients. Twenty-five percent of the severe cases were nonsurvivors. While there was an increase in the levels of inflammatory markers in all severe COVID-19 patients, they were significantly higher in nonsurvivors compared to survivors. Several studies have been conducted to ascertain the relationship between inflammatory markers and the overall outcome of patients with COVID-19 disease. Alroomi showed that ferritin independently predicts in-hospital mortality in Kuwaiti population [12]. A retrospective study of Huang using a large sample size of 1751 Chinese patients showed that LDH associated with higher mortality risk [13]. In contrast, the meta-analysis of Martha concluded that LDH was associated with poor prognosis in COVID-19 patients [14]. Ahmeidi showed that elevation in serum inflammatory marker CRP may be indicative of COVID-19 infection severity and mortality and suggested that these parameters may predict COVID-19 severity [15]. However, their study was limited by inadequate sample size and study design. Yet in another study, El-Shabrawy showed that CRP/albumin ratio predicted 30-day mortality in COVID-19 patients [16].

This study showed that COVID-19 male patients had significantly higher values of ferritin, urea, and creatinine compared to female counterparts. Previously, Gandini showed that higher ferritin levels in males could predict worse outcome in male patients [17]. This is contradictory to the study of Chen, where female COVID-19 patients had significantly higher ferritin levels than male counterparts [18]. Further, a retrospective analysis of 12,413 COVID-19 patients showed that serum creatinine and blood urea nitrogen (BUN) levels are higher in males than females [19].

In the current study, SpO_2_ was reduced in COVID-19 patients and showed a negative correlation with LDH and ferritin. Though no previous studies reported the correlation of ferritin with SpO_2_, Poggiali et al. revealed by retrospective observation study that CRP (*r* = 0.55, *p* value < 0.0001) and LDH (*r* = 0.62, *p* value < 0.0001) showed strong inverse correlation with the respiratory performance (PaO2/FiO2) [20]. Significant elevation of potassium, urea, and creatinine levels in severe COVID-19 nonsurvivors compared to survivors who had normal levels of urea and creatinine, positive correlation of LDH and ferritin with urea and creatinine levels, and positive correlation of LDH with serum potassium levels are indicative of inflammation-mediated renal failure among nonsurvivors. This is in line with the study of Ng et al. among hospitalized COVID-19 patients, which showed higher rate of mortality among patients with end-stage kidney disease than those without this disease [21].

The ROC analysis shows LDH and ferritin can be predictors of mortality in severe COVID-19 patients. Despite showing strong correlation with LDH and ferritin, ROC analysis could not infer the predictive role of CRP in COVID-19 mortality.

The current study had some limitations. The study is a single-center study and used cohort study design without controls and nonsevere patients. Multi-center study with larger sample size must be done including controls. In addition to LDH, CRP, and ferritin, other inflammatory markers such as interleukin-6 (IL-6) and procalcitonin could be correlated with disease severity to predict COVID-19 mortality [22, 23]. Integration of neutrophil/lymphocyte (N/L) ratio and CRP may lead to improved prediction [24], which needs to be addressed in future studies. Intravenous heparin administration in severe COVID-19 patients was done as a line of management to prevent mortality. However, further studies correlating lines of management with the clinical data of ventilated patients are warranted.

COVID-19 infection is accompanied by vigorous immune and inflammatory response that causes severe lung damage to limit the entry of oxygen to the bloodstream, resulting in long-term breathlessness and severe complications including renal failure. Inflammatory markers (ferritin/LDH) could be useful as a predictor for COVID-19 mortality and respiratory failure and could help the physicians to discern at-risk COVID-19 patients to facilitate early treatment. Elevated LDH increases the odds of severe COVID-19 disease and mortality among ICU-admitted patients.

## Conclusion

LDH and ferritin can predict mortality in severe COVID-19 patients. Special attention to elevated levels of these inflammatory markers will enable better management.

## Abbreviations

ALP: Alkaline phosphatase
ALT: Alanine transaminase
AST: Aspartate transaminase
AUC: Area under the curve
CRP: C-reactive protein
Hb: Hemoglobin
ICU: Intense care unit
IL-6: Interleukin-6
LDH: Lactate dehydrogenase
N/L: Neutrophils/lymphocytes
RBS: Random blood sugar
ROC: Receiver operating characteristic
SpO_2_: Oxygen saturation
